# The sooner, the better: ROS, kinases and nutrients at the onset of the damage response in *Drosophila*

**DOI:** 10.3389/fcell.2022.1047823

**Published:** 2022-10-24

**Authors:** Florenci Serras

**Affiliations:** Department of Genetics, Microbiology and Statistics, Faculty of Biology, and Institute of Biomedicine of the University of Barcelona, University of Barcelona, Barcelona, Spain

**Keywords:** apoptosis, regeneration, ASK1 (apoptosis signal regulating kinase 1), p38, wound healing, JNK, ROS, reactive oxygen species

## Abstract

One of the main topics in regeneration biology is the nature of the early signals that trigger the damage response. Recent advances in *Drosophila* point to the MAP3 kinase Ask1 as a molecular hub that integrates several signals at the onset of regeneration. It has been discovered that reactive oxygen species (ROS) produced in damaged imaginal discs and gut epithelia will activate the MAP3 kinase Ask1. Severely damaged and apoptotic cells produce an enormous amount of ROS, which ensures their elimination by activating Ask1 and in turn the pro-apoptotic function of JNK. However, this creates an oxidative stress environment with beneficial effects that is sensed by neighboring healthy cells. This environment, in addition to the Pi3K/Akt nutrient sensing pathway, can be integrated into Ask1 to launch regeneration. Ultimately the activity of Ask1 depends on these and other inputs and modulates its signaling to achieve moderate levels of p38 and low JNK signaling and thus promote survival and regeneration. This model based on the dual function of Ask1 for early response to damage is discussed here.

## Introduction

Regeneration, involving the repair or replacement of body parts, is widespread across the metazoans (Bely and Nyberg, 2010). The extent of regeneration varies among phyla, ranging from complete body parts to small pieces of damaged cells. Regardless of their regenerative capacity, different animal models, from worms to mammals, are being used in laboratories worldwide to shed some light on the basic principles of regeneration (Goldman and Poss, 2020). Research using animal models has generated several breakthroughs and has accumulated a vast amount of knowledge about the factors necessary for wound healing, regenerative proliferation, and re-patterning (Reuter et al., 2019). A definition of regeneration reminiscent of T.H. Morgan (Morgan, 1901) entails two scenarios in metazoans: (a) reparative or traumatic regeneration and (b) a physiological or homeostatic scenario, including cell turnover and tissue or epithelial lining wear and tear. In fact, optimal health is largely dependent upon tissue homeostasis, which involves cell replacement, regeneration, and tissue repair.

However, little is known about how tissues sense damage to initiate a cellular response. This aspect can be studied in model organisms where damage and its response can be reproducibly traced in small populations of cells and where genetic manipulation is possible, such as in *Drosophila* (Fox et al., 2020). How cells know when the regeneration machinery must be ignited is the subject of this paper, and in particular, I will focus on recent contributions that have unveiled the early dialog between damaged cells and healthy regenerating cells in *Drosophila*.

*Drosophila* imaginal discs are a model for tissue regeneration, as they undergo compensatory proliferation upon damage (Fan and Bergmann, 2008; Martin et al., 2008; Perez-Garijo et al., 2009; Smith-Bolton et al., 2009; Bergantiños et al., 2010; Herrera et al., 2013). Moreover, imaginal discs, like the mammalian liver, are epithelia that can heal and regenerate to their original size following removal of part of their mass, implying that some form of memory is retained in these organs (Hariharan and Serras, 2017). The wing imaginal discs, which are easily accessible, permit the manipulation and monitoring of both fast responses in the larval tissue and delayed responses in the adult tissue.

## How is damage sensed?

Upon damage, the internal and external microenvironments can act as stressors for the cell. Stressors can affect the ionic balance of the cell membranes, change the permeability of the mitochondria and cause DNA damage. For example, injuries can produce intracellular Ca^2+^ release from the endoplasmic reticulum Ca^2+^ stores downstream of the inositol-3-phosphate receptor, which in turn requires gap junctions for intercellular propagation during wound healing (Razzell et al., 2013; Narciso et al., 2015; Restrepo and Basler, 2016). Also, ROS produced as byproducts of mitochondrial dysfunction generate oxidative stress that can also propagate to healthy neighbor cells (Serras, 2016). There are different sources of ROS in the cell. The evolutionary conserved membrane bound NAPH-oxidases (e.g. Duox, Nox) are specialized ROS producers and are involved in signaling (Vermot et al., 2021). ROS are also produced during mitochondrial electron transport or oxidation reactions. The free radical superoxide (O_2_^−^), which predominates in the mitochondria, is the result of the reduction by one electron to oxygen. Two-electron reactions that reduce oxygen to hydrogen peroxide (H_2_O_2_) also occur in mitochondria (Finkel, 2011). ROS, which have generally been considered to be deleterious, are now emerging as active participants in cell signaling events (Finkel, 2011). For example, H_2_O_2_ is required for inflammatory cell recruitment (Niethammer et al., 2009; Moreira et al., 2010; Hunter et al., 2018; Niethammer, 2018). ROS produced after *Xenopus* tail or zebrafish fin lesions are required for regeneration (Gauron et al., 2013; Love et al., 2013; al Haj Baddar et al., 2019). ROS can rescue regeneration in Erk-deficient planarians (Jaenen et al., 2021). H_2_O_2_ is generated by the activation of NADPH oxidases (DUOX) and is important for rapid recruitment of pro-inflammatory cells to the wound (Razzell et al., 2013; Narciso et al., 2015). ROS and Ca^++^ production are interrelated as blocking the calcium waves or flashes produced after damage inhibits H_2_O_2_ release at the wound site, suggesting that the wound-induced Ca^++^ activates DUOX, likely *via* an EF-hand calcium-binding motif that, in turn, produces H_2_O_2_ (Razzell et al., 2013; Khan et al., 2017). Damaged cells can propagate H_2_O_2_ extracellularly and aquaporins might then act as conduits that are needed for these extracellular ROS to be channeled into the nearby healthy cells (Thiagarajah et al., 2017; Dhawan et al., 2021). Several lines of evidence show that interfering with Ca^++^ flashes or with oxidative stress alters wound healing and tissue repair (Razzell et al., 2013; Santabárbara-Ruiz et al., 2015; Restrepo and Basler, 2016; Brock et al., 2017; Khan et al., 2017). Therefore, these mechanisms operate as chemical alerts to sense damage and eventually activate regeneration.

In summary, the capacity to sense damage by Ca^++^ or ROS is a primary hallmark of tissue regeneration. ROS produced by various redox metabolic reactions have recently emerged as active participants in cell signaling events that spark regeneration.

### JNK and p38: two MAP kinases that respond to ROS generated by cell damage

ROS act as second messengers to activate redox-sensitive signals, including the stress activated MAP kinases Jun-N Terminal kinase (JNK) and p38 (Droge, 2002; McCubrey et al., 2006; Jiang et al., 2011; Sato et al., 2014). In mammals, apoptosis enhances the activation of p38, resulting in Wnt3 transcription, a signal required for regeneration (Ankawa et al., 2021). In *Drosophila,* p38 and JNK signaling pathways respond to ROS and foster regeneration (Matsuzawa et al., 2005; Shi et al., 2014; Santabárbara-Ruiz et al., 2015; Verghese and Su, 2016; Brock et al., 2017; Khan et al., 2017; Pérez et al., 2017; Diwanji and Bergmann, 2018; Worley et al., 2018; Patel et al., 2019; Evans et al., 2022).

**FIGURE 1:**
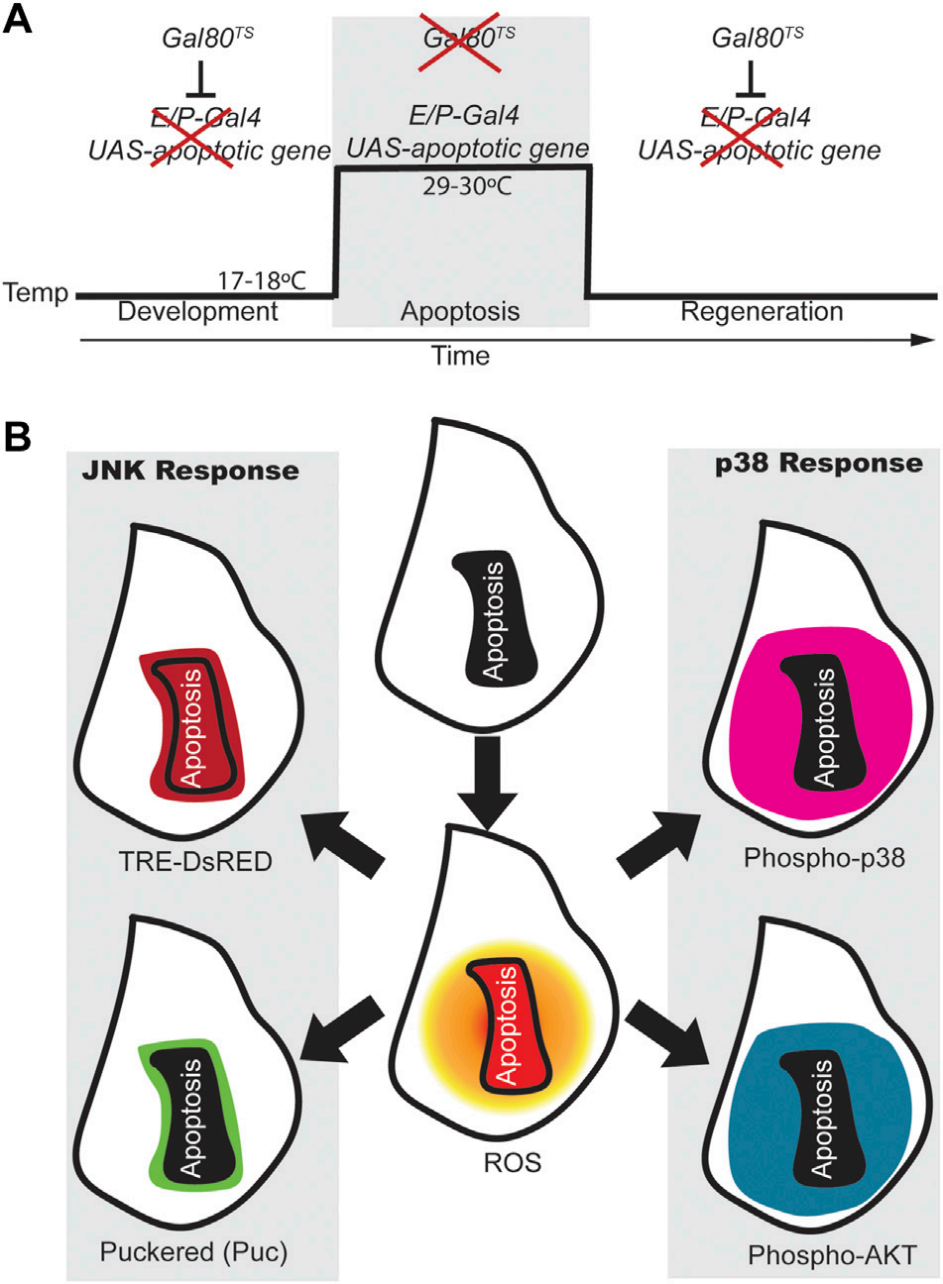
ROS-dependent responses in early regeneration. **(A)** Design for genetic induction of cell death (genetic ablation). *Drosophila* strains carrying tissue specific (enhancer/promoter) expression of Gal4 (*E/P-Gal4*) will allow the transcription of the pro-apoptotic gene cloned downstream of the UAS sequence when Gal80 is inhibited (at 29–30°C). However, at 18°C the temperature sensitive Gal80 is active and blocks Gal4 which results in regeneration of the apoptotic tissue. **(B)** Scheme of wing imaginal discs showing the activity of some of the early responding signals after apoptosis. After genetic ablation, apoptotic cells produce high levels of ROS (Red in the central disc), which spreads to cells surrounding the apoptotic domain (shaded orange circle); Apoptotic cells and few non-apoptotic cells nearby, show expression of the JNK reporter TRE-DsRed (dark red). The *puckered* phosphatase (*puc* in green) is transcribed downstream JNK mainly in cells that are nearby the apoptotic zone. P-Akt and P-p38 respond in a broader domain and both found in surrounding the dead domain. Apoptotic cells are outlined with a black line in the center of each disc or with a solid black background.

Because imaginal discs do not show significant apoptosis, the ectopic activation of pro-apoptotic genes in discrete zones is a suitable technique for neatly testing tissue recovery (Smith-Bolton et al., 2009; Bergantiños et al., 2010). This approach is less laborious than traditional surgical methods and can, therefore, be incorporated into large-scale genetic studies. It is based on the expression of the yeast transcription factor Gal4 targeted at discrete zones of the epithelium using tissue specific regulatory elements and also at a UAS that drives the expression of pro-apoptotic genes. With these genetic tools, the binding of Gal4 to the UAS will result in apoptosis in discrete zones of the tissue. The activity of Gal4 can be inhibited by a temperature-sensitive allele of Gal80, a Gal4 repressor. Genetic ablation is achieved by a temperature shift (from 17°C to 30°C) for a period of several hours during third instar larval development (Figure 1A).

When apoptosis is induced in imaginal discs, stress factors stimulate the pro-apoptotic role of JNK, resulting in a loop that ensures apoptosis (Shlevkov and Morata, 2012; Wells and Johnston, 2012). However, in addition to the pro-apoptotic role of JNK, proliferative and developmental roles have been widely reported (Pinal et al., 2019). Reporters of JNK activity, such as TRE-DsRed (Chatterjee and Bohmann, 2012), have been detected in cells that undergo apoptosis, but also in cells adjacent to the apoptotic zone, (Santabárbara-Ruiz et al., 2015) (Figure 1B). The phosphatase *puckered* (*puc*) is expressed downstream of the JNK pathway and acts as a negative regulator of the pathway (McEwen and Peifer, 2005). Puc is found at the edge of injured or apoptotic zones (Bosch et al., 2005, 2008; Smith-Bolton et al., 2009; Bergantiños et al., 2010; Santabárbara-Ruiz et al., 2015) (Figure 1B). In addition, blocking ROS production or mutants in the JNK pathway prevent regeneration (Smith-Bolton et al., 2009; Bergantiños et al., 2010; Fogarty et al., 2016; Khan et al., 2017).

Another MAP kinase that controls cellular responses to stress is p38. Phospho-p38, a reporter of p38 signaling, accumulates considerably in the imaginal disc, particularly in cells that surround the apoptotic zone (Figure 1B). Chemical antioxidants and genetic ROS scavengers interfere with the activation of p38; and loss-of-function of p38 signaling results in impaired regeneration (Santabárbara-Ruiz et al., 2015). Interestingly, the domain of *puc* cells is restricted to a few cells at the edge of the apoptotic zone, whereas increased p38 activity is much more extensive (Figure 1B). This observation is reminiscent of the antagonism between p38 and JNK (Wagner and Nebreda, 2009). In *Drosophila* gut, the interaction between p38 and its major target MK2 ensures proper stress response by keeping JNK activity low to avoid apoptosis (Seisenbacher et al., 2011).

### The link between ROS production and JNK or p38 activation

There are two crucial questions that need to be addressed to understand the onset of repair. First, how is the production of ROS linked to JNK and p38 activity? And second, as the two MAPKs can promote apoptosis, how do JNK and p38 avoid killing the cells and be essential for regeneration? Recent advances have shed some light on these issues.

A key molecule in both instances is the apoptosis signal regulating kinase-1 (ASK1). Among the MAP3 kinases that operate upstream from JNK and p38, ASK1 is particularly sensitive to oxidative stressors (Takeda et al., 2006; Shiizaki et al., 2013; Sakauchi et al., 2017). ASK1 is inhibited when bound to thioredoxin (TRX) at the N-terminal region and to 13-4-4 protein close to the C-terminus (Saitoh et al., 1998; Zhang et al., 1999). Upon oxidative stress, these inhibitory proteins dissociate from the Ask1 complex and, thereby, ASK1 oligomerizes, autophosphorylates, recruits partners and becomes active (Bunkoczi et al., 2007; Takeda et al., 2008; Obsil and Obsilova, 2017). A threonine-rich catalytic domain is important for ASK1 signaling and can trigger JNK- and p38-dependent apoptosis (Ichijo et al., 1997; Tobiume et al., 2001, 2002; Bunkoczi et al., 2007).

Little is known about the partners and mechanism of action of the *Drosophila* Ask1. Ask1 can induce apoptosis in a JNK-dependent manner (Kuranaga et al., 2002). But loss of function of Ask1 impairs regeneration, which indicates that it has functions other than those related to apoptosis (Patel et al., 2019; Santabárbara-Ruiz et al., 2019). The activation of p38 (phospho-p38) mainly occurs in non-apoptotic cells and is Ask1 dependent (Patel et al., 2019; Santabárbara-Ruiz et al., 2019).

In addition to the autophosphorylation of Thr-rich sites, the phosphorylation of various Ser residues by other kinases also regulates mammalian ASK1 signaling activity (Zhang et al., 1999, 2005; Kim et al., 2001; Fujii et al., 2004). Interestingly, phosphorylation of human ASK1 Ser83 by Akt is thought to attenuate Ask1 activity and inhibit apoptosis (Kim et al., 2001; Zhang et al., 2005). *Drosophila* lacks the vertebrate N-terminal region that includes Ser. However, position 83 of *Drosophila* Ask1 is occupied by another Ser residue (Ser174 in human ASK1) embedded in a consensus that is highly conserved from sponges to mammals and that can be phosphorylated by Akt (Santabárbara-Ruiz et al., 2019). Amino-acid substitution of this Ser83/174 impedes p38 phosphorylation and regeneration, suggesting that at least in flies, Ask1 Ser83/174 is key for survival, skipping apoptosis and promoting repair (Santabárbara-Ruiz et al., 2019). Remarkably, healthy cells near apoptotic cells not only show increased phosphorylation of p38 but also a ROS-dependent increase in phosphorylated Akt, also known as Protein Kinase B (PKB) (Santabárbara-Ruiz et al., 2019) (Figure Fig1B). Thus, ROS have a dual function, one to activate Ask1 by removing Trx and the other to promote the activity of Akt. The Akt pathway can be enhanced through inhibition of the counteracting PTEN phosphatase. Interestingly, hydrogen peroxide oxidizes and inactivates human PTEN through disulfide bond formation between the catalytic domain Cys-124 and Cys-71 residues (Lee et al., 2002).

In summary, phosphorylation of Ask1 by Akt promotes survival, perhaps by keeping JNK at low levels and thus avoiding JNK-induced apoptosis, and by producing moderate levels of phospho-p38. These low JNK and moderate p38 levels set the scene for triggering tissue repair in healthy cells and avoiding apoptosis. Moreover, Akt-dependent phosphorylation of the Ser83/174 residue is required for p38-activity and for low JNK activity. Thus, this Ser83/174 residue must be key for swapping between p38 or JNK, and therefore between survival or apoptosis.

The adult *Drosophila* gut, a stem cell-based regenerating tissue, has many structural similarities to the human intestine and is the subject of intensive research that has been reviewed elsewhere (Colombani and Andersen, 2020; Zhang and Edgar, 2022). Remarkably, p38 is activated in enterocytes upon damage, and Ask1 acts upstream from p38 in response to numerous stressors, among them ROS produced by the NAPH oxidase NOX. This NOX/Ask1/P38 module in enterocytes acts as the signal for Upd cytokines to induce intestinal stem cells to proliferate, repair damage and regenerate the gut (Patel et al., 2019). Moreover, p38 signaling protects enterocytes from JNK-induced apoptosis after chronic stress (Seisenbacher et al., 2011).

### Kinases and nutrients

Akt mediates phosphatidylinositol 3-kinase (Pi3K) to modulate different signals that foster proliferation and survival and prevent apoptosis. One of the canonical signals upstream from Pi3K/Akt is the insulin/insulin-like growth factor signaling pathway (IIS) that, upon activation of its tyrosine-kinase receptor, PIP2 is converted to PIP3 by Pi3K (Revathidevi and Munirajan, 2019). PIP3 recruits Akt to the plasma membrane and binds to phosphoinositide-dependent kinase-1 (PDK1), which in turn phosphorylates Akt. The conserved IIS and the target of rapamycin (TOR) pathways have a central role in metabolic homeostasis, stress response, growth, and aging, in flies, worms and mammals (Partridge et al., 2011).

One of the central mechanisms that organisms use to sense nutrients and their own nutritional status is the highly evolutionarily conserved insulin/insulin-like growth factor signaling (IIS) and the target of rapamycin (TOR) pathways (Flatt and Partridge, 2018). The decreased activity of these pathways can improve health and delay aging (Johnson et al., 2013; Flatt and Partridge, 2018). However, reduced activity of the IIS and TOR pathways can impair wound healing in flies and mice (Squarize et al., 2010; Kakanj et al., 2016). Thus, it is conceivable that the mechanisms that sense the nutritional status of the organism are key in sensing damage and therefore in the onset of regeneration. Indeed, it has recently been found that nutrient restriction blocks regeneration in similar phenotypes such as downregulation of Pi3K/Akt or in the mutated Ser83 of Ask1 (Santabárbara-Ruiz et al., 2019; Esteban-Collado et al., 2021). More importantly, it has also been found that the anomalous regeneration resulting from nutrient restriction or from mutated Ask1 Ser83 can be rescued by ectopic activation of p38, but not of JNK (Esteban-Collado et al., 2021). These observations demonstrate that the function of Pi3K/Akt in regeneration is channelled through p38 rather than JNK.

## Discussion

**FIGURE 2:**
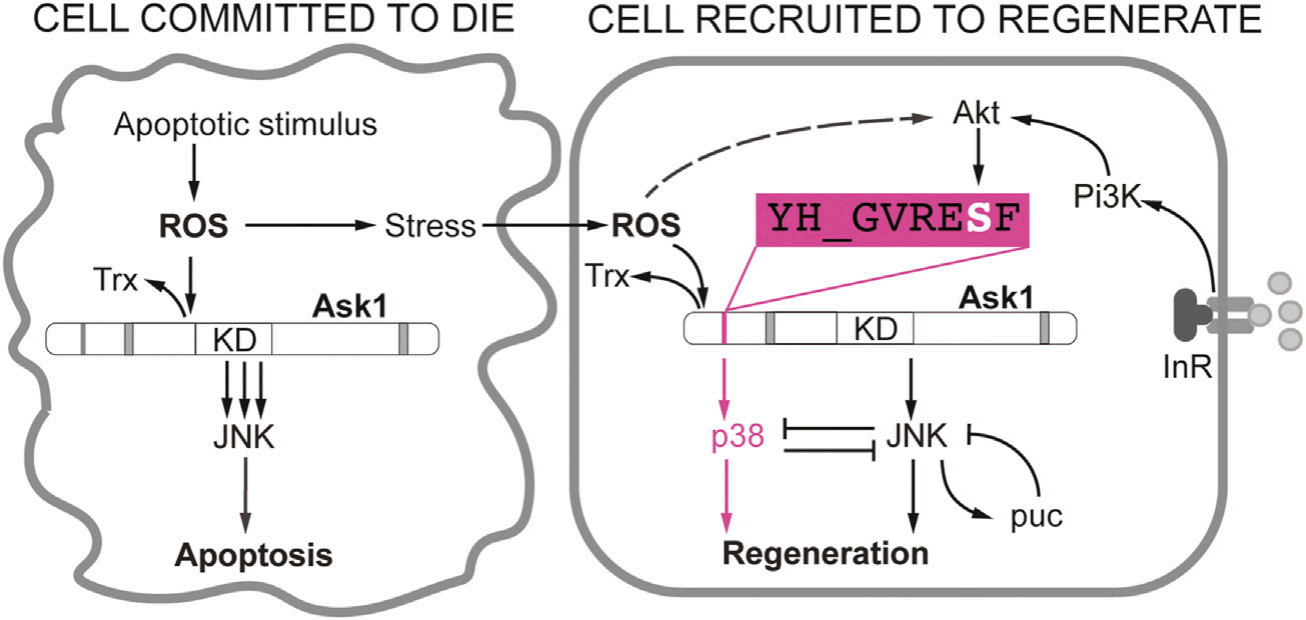
Different requirement of JNK and p38 in *Drosophila* wing disc cells during apoptosis and regeneration. Apoptotic stimulus produces ROS that promote the dissociation of the thioredoxin (Trx) from Ask1. As a consequence, the activity of the Ask1 kinase domain is enhanced and JNKK will be phosphorylated and eventually the cell will irreversibly enter into apoptosis (Cell committed to die). The adjacent healthy cells will be sensitive to propagated ROS from the apoptotic cell, albeit in much lower levels, and moderately activate Ask1 (Cell recruited to regenerate). Ask1 can activate JNK in low levels or transiently until *puckered* (*puc*) attenuates JNK. In addition, the JNK-p38 antagonism could also attenuate JNK in the regenerating cell. This is key to maintain the viability of the regenerating cell. Activation of the Pi3K/Akt by nutrients or insulin signaling will result in phosphorylation of the Ser residue of YH_GVRESF Ask1 consensus. This signal is crucial for the activation of p38 but not JNK. Akt is also sensitive to ROS, likely through PTEN inactivation (dotted line).

In the last two decades several types of *Drosophila* epithelia, from embryo to adult, have contributed to our understanding of the early signals that respond to damage in an organism. A central feature is Ask1, a kinase initially associated with promoting cell death that has now emerged as being pivotal in regeneration. We foresee Ask1 as a signaling hub that integrates early moderate Ca^++^/ROS signals and phospho-Akt survival signals to divert Ask1 towards the p38-regeneration response (Figure 2). In contrast, high ROS or pro-apoptotic signals will act on the Ask1 hub to steer it towards JNK-dependent apoptosis (Figure 2). In addition to ROS and IIS/Pi3K/Akt, the decision to survive or not may require other molecules interacting with Ask1. Various signaling proteins such as the TNF receptor associated factors (TRAF) can bind to Ask1 and modulate its function (Nishitoh et al., 1998; Matsuzawa et al., 2005; Fujino et al., 2007).

JNK has a variety of functions other than promoting apoptosis (Martin-Blanco et al., 1998, 2000; Zeitlinger and Bohmann, 1999; Pastor-Pareja et al., 2004; Muñoz-Descalzo et al., 2005; Gettings et al., 2010; Martín et al., 2017; Pinal et al., 2019). JNK’s function in early regeneration events will strongly depend on its levels or perhaps on how long these are sustained. In *Drosophila* discs, constitutive activation of JNK results in apoptosis (Igaki, 2009; Shlevkov and Morata, 2012). But in physical injuries, JNK is activated at the wound edges (Bosch et al., 2005, 2008; Lee et al., 2005; Mattila et al., 2005; Blanco et al., 2010; Diaz-Garcia and Baonza, 2013; Ahmed-de-Prado et al., 2018). Reporters of JNK (i.e. TRE-dsRed) are expressed earlier than the phosphatase *puc* (i.e. puc > GFP) (Santabárbara-Ruiz et al., 2015). In addition, the cell lineage of these *puc* positive cells has shown that most of the reconstructed disc derives from *puc* positive cells (Bosch et al., 2008). The presence of *puc* positive cells indicates that JNK has been activated and dampened down, likely to restrict JNK to beneficial levels.

An exciting suggestion would be that JNK responds rapidly to the first flashes of ROS or Ca^++^. But once the phospho-Akt diverts Ask1 towards the survival pathway, JNK activity is restricted to protecting the tissue from further damage. This is an attractive hypothesis as it implies that the onset of stress-induced repair has two phases: one more dependent on JNK and a second on p38. Further research will be needed to elucidate whether the activity of these kinases overlaps.

The transcriptional signature downstream of the damage response includes the program for regenerative proliferation and repatterning (Vizcaya-Molina et al., 2018; Harris et al., 2020; Worley et al., 2022) and the identification of the blastema signature (Worley et al., 2022). In addition, JNK and p38 MAP kinases target the unpaired (Upd) cytokines (the *Drosophila* members of the interleukin-6 family), which are capable of activating the JAK/STAT pathway for growth control (Pastor-Pareja et al., 2008; Jiang et al., 2009; Diaz-Garcia and Baonza, 2013; Katsuyama et al., 2015; Santabárbara-Ruiz et al., 2015; la Fortezza et al., 2016; Verghese and Su, 2016; Ahmed-de-Prado et al., 2018; Worley et al., 2018; Patel et al., 2019). Also, non-apoptotic JNK activity in regeneration is driven by, for example, Wg/Wnt signaling (Smith-Bolton et al., 2009; Harris et al., 2016; Gracia-Latorre et al., 2022), Hippo signaling (Grusche et al., 2011; Sun and Irvine, 2011; Repiso et al., 2013; Meserve and Duronio, 2015; Ruiz-Romero et al., 2015), *Gadd45*, involved in DNA repair (Camilleri-Robles et al., 2019), and *Ets21c*, a JNK-dependent transcription factor key for disc blastema cells (Worley et al., 2022). Further research will be necessary to clarify whether the genetic response to JNK and p38 differs. In addition, studies are needed to explore whether this mechanism of sensing damage has been conserved through evolution.
